# Glyphosate’s Synergistic Toxicity in Combination with Other Factors as a Cause of Chronic Kidney Disease of Unknown Origin

**DOI:** 10.3390/ijerph16152734

**Published:** 2019-07-31

**Authors:** Sarath Gunatilake, Stephanie Seneff, Laura Orlando

**Affiliations:** 1 Health Science Department, California State University Long Beach, Long Beach, CA 90840, USA; 2 Computer Science and Artificial Intelligence Laboratory, Massachusetts Institute of Technology, Cambridge, MA 02139, USA; 3 Environmental Health Department, Boston University School of Public Health, Boston, MA 02118, USA

**Keywords:** CKDu, kidneys, glyphosate, paraquat, pesticides, glycine, dehydration

## Abstract

Chronic kidney disease of unknown etiology (CKDu) is a global epidemic. Sri Lanka has experienced a doubling of the disease every 4 or 5 years since it was first identified in the North Central province in the mid-1990s. The disease primarily affects people in agricultural regions who are missing the commonly known risk factors for CKD. Sri Lanka is not alone: health workers have reported prevalence of CKDu in Mexico, Nicaragua, El Salvador, and the state of Andhra Pradesh in India. A global search for the cause of CKDu has not identified a single factor, but rather many factors that may contribute to the etiology of the disease. Some of these factors include heat stroke leading to dehydration, toxic metals such as cadmium and arsenic, fluoride, low selenium, toxigenic cyanobacteria, nutritionally deficient diet and mycotoxins from mold exposure. Furthermore, exposure to agrichemicals, particularly glyphosate and paraquat, are likely compounding factors, and may be the primary factors. Here, we argue that glyphosate in particular is working synergistically with most of the other factors to increase toxic effects. We propose, further, that glyphosate causes insidious harm through its action as an amino acid analogue of glycine, and that this interferes with natural protective mechanisms against other exposures. Glyphosate’s synergistic health effects in combination with exposure to other pollutants, in particular paraquat, and physical labor in the ubiquitous high temperatures of lowland tropical regions, could result in renal damage consistent with CKDu in Sri Lanka.

## 1. Introduction

Starting in the early 1990s, an epidemic of chronic kidney disease (CKD) was discovered among rice paddy farmers, primarily young men, in the North Central province of Sri Lanka [1,2]. The disease now affects a total population of over 150,000 with estimated annual deaths of 5,000, with a doubling of the disease every 4 or 5 years [3]. The most unique feature of this CKD is that its etiology does not include commonly known risk factors such as diabetes mellitus, hypertension and glomerular nephritis. Because of this, it is often referred to as chronic kidney disease of unknown origin or unknown etiology (CKDu). The prevalence of CKDu is 15.1–22.9% in some Sri Lankan districts [4]. Similar epidemics of CKDu have been reported mostly among sugarcane workers along the Pacific coast of Mexico and Central America (Mesoamerican nephropathy), and agricultural workers in Andhra Pradesh in India (Uddanam epidemic nephropathy). In El Salvador, it is the second leading cause of death, with a 30.6% increase between 2007 and 2017 [5]. Other countries affected by the disease include Guatemala, Costa Rica, Honduras, and Egypt [6,7].

Several researchers and organizations, including the World Health Organization (WHO), have conducted studies to pinpoint the cause of CKDu in Sri Lanka. However, none of these studies were conclusive in their findings. The purpose of this paper is to review the current literature on the causative factors related to the CKDu in Sri Lanka with a view toward synthesizing these findings into a single model that could explain the etiology of the disease according to the present status of our knowledge. Some of the possible biochemical mechanisms involved in the pathogenesis of CKDu and other adverse health effects will also be discussed. 

Some recent studies have attempted to make clinical and histopathological comparisons between Mesoamerican nephropathy (MeN) and Sri Lankan agricultural nephropathy (CKDu). Like CKDu, MeN has an unknown etiology. Chronic interstitial nephritis and glomerular sclerosis are the most common pathologic findings in both types of CKDs [8]. These types of pathological changes are mostly evident in occupational and/or environmental exposures. Similar findings have been reported following excessive exposure to lead, cadmium, and aristolochic acid [9]. The herbicide glyphosate has been implicated as a key factor in both MeN [10] and CKDu in Sri Lanka [11,12]. A pathological picture consistent with a toxic nephropathy was present in kidney biopsies from both El Salvador and Sri Lanka [9]. In a more recent study, Wijkström et al. (2018) [13] found that the morphology and biochemical characteristics of CKDu patients in Sri Lanka have many resemblances with the MeN epidemic in Central America, indicating a similar diagnostic entity and possibly a similar etiology. With these fairly consistent pathological findings of chronic interstitial nephritis and glomerular sclerosis, there is enough agreement that the Sri Lankan CKDu is a toxic nephropathy [11,14,15,16,17,18,19]. However, there is much less agreement among researchers as to what combination of occupational exposures and environmental toxicants manifest to CKDu. 

In this paper, we will develop a hypothesis that CKDu in Sri Lanka is a consequence of the synergistic toxicity of glyphosate in combination with a number of different toxic agents, including paraquat, excessive fluoride and phosphate exposure, heavy metals, surfactants and pathogenic toxins, along with dehydration. It has been argued that glyphosate-based formulations are much more toxic than glyphosate in isolation [20]. The formulations contain additives such as polyoxyethylenamines (POEA) and Quaternary ammonium compounds (QAC), which were shown in toxicity studies to be considerably more acutely toxic than glyphosate itself. They also detected toxic metals such as arsenic, chromium, lead and nickel in the formulations, which are known nephrotoxins.

While it had been widely believed that glyphosate is relatively nontoxic to humans, recent evidence has dramatically changed that perception. The International Agency for Research on Cancer (IARC) labeled glyphosate a “probable carcinogen” in March, 2015, and the state of California followed suit by listing it as a “probable carcinogen” requiring labeling. In a period of less than a year from August, 2018 to June, 2019, three successful California lawsuits involving a causal link between glyphosate and non-Hodgkin’s lymphoma resulted in jury awards of over two billion U.S. dollars. This caused a dramatic drop in the value of Bayer’s stock, since Bayer had acquired Monsanto not long before these lawsuits were litigated. In July, 2019, Austria became the first European country to ban glyphosate for all uses [21].

We argue here that glyphosate, even without the added formulants, has a unique insidious mechanism of toxicity that involves substituting erroneously for the coding amino acid glycine during protein synthesis. This leads to disruption of proteins that are critical for the detoxification and removal of other environmental chemicals, causing them to be much more nephrotoxic than they would otherwise be. Nephropathy at the nexus of glyphosate, paraquat, and dehydration presents a path of exploration for future research into this global health crisis among agricultural workers.

## 2. Environmental Condition of People with Chronic Kidney Disease of Unknown Etiology 

Given the occurrence of CKDu only over the past thirty years and nearly always in agricultural communities, it is highly likely that the factors involved are a result of human activities related to agriculture and climate change. Some researchers emphasize the agricultural component by referring to the disease as Chronic Interstitial Nephritis in Agricultural Communities or CINAC [22]. Nevertheless, the disease targets the proximal renal tubules of the kidneys of people who live in hot climates that are getting hotter [23] and more polluted [24,25].

One possible result of the accumulation of multiple toxins in agricultural areas is the observation that children in CKDu endemic regions are also manifesting with early renal damage [26]. CKDu risk factors consistently identified in multiple studies include male sex, being a farmer, using and/or spraying pesticides—often without adequate personal protective equipment (PPE), drinking well water at home or in the field, and having a family member with renal dysfunction [27,28,29,30]. Poverty, lack of education, inadequate medical attention, and limited or no access to safe drinking water also have been identified as a constellation of contributory factors [31]. Although many authors suggest a multifactorial etiology [3,27,28,29], new evidence is beginning to accumulate on the role of drinking water and irrigation systems for paddy cultivation as a vehicle for toxicants that may contribute to CKDu in Sri Lanka [32,33]. Most paddy lands in the CKDu-affected area are irrigated by an ancient cascade system of tanks. Abeysingha and colleagues provide a detailed account of how the design of the cascade irrigation system and the adverse changes that took place to the ecosystem with the introduction of new commercial practices of agriculture (mostly the use of agrochemicals) in the CKDu endemic area have contributed to its rapid spread [34]. 

A revealing anthropological study that interviewed 200 participants in the CKDu-affected areas indicated that 94% of those from agricultural settlements, 86% from old villages and 80% from the Vedda (indigenous) villages view kidney disease as being caused by drinking polluted water from dug wells and tube wells [35]. The impressions of these villagers were scientifically confirmed by the discovery of early renal damage among children living in the region of highest CKDu burden [26]. Jayasekera et al. have shown that the emerging new foci of CKDu are located in villages below the level of the reservoirs/canals. This may indicate the possibility that the irrigated water is draining into the shallow wells of the households, which is the main source of drinking water [1,32].

Multiple research studies have analyzed the content of calcium, magnesium, fluoride and other heavy metals such as arsenic, cadmium, iron, lead and aluminum, in the irrigated water reservoirs and the shallow wells that were once used as a source of drinking water by the farmers [36,37,38]. Most of these wells are now abandoned because of an unacceptable musty taste (described as “Kivul” by villagers). Jayasumana et al. have shown that drinking well water and a history of drinking water from abandoned wells are important risk factors for CKDu (with Odds Ratios 2.52, (95% CI 1.12–5.70) and 5.43, (95% CI 2.88–10.26) respectively) [33]. It was demonstrated in the same study that 94% of the abandoned wells contained more than 1 μg/L of glyphosate while 31% of the currently serving wells contained glyphosate above the level of 1 μg/L. They also reported that the subjects who sprayed glyphosate were four times more likely to develop CKDu compared to those without such a history. 

The only other study that tested for glyphosate in well water was a study conducted by the World Health Organization (WHO) [36]. In addition to glyphosate, this WHO study found cadmium (a known nephrotoxic metal), arsenic and lead, along with low (depleted) selenium levels. The study also reported that 65 percent of the subjects excreted glyphosate in urine and another 28 percent excreted its metabolite, aminomethylphosphonic acid (AMPA). The authors stated that “simultaneous exposure of people to heavy metals and nephrotoxic pesticides may be a contributory factor in the pathogenesis and progression of CKDu.” It has been demonstrated that the main source of these heavy metals in drinking water is from fertilizer tainted with these metals [39]. In addition to the abundant use of contaminated fertilizer, Sri Lanka has one of the highest pesticide use rates, ranking 4th in Asia [40].

According to the same author, who works for the Sri Lankan Registrar of Pesticides, 33 percent of vegetable samples were contaminated with pesticide residues. These pesticides included diazinon, phenthoate, prothiofos, oxyfluorfen, tebuconazole, and also the already banned pesticide chlorpyrifos. It is interesting to note that this same study did not test for glyphosate residues, even though this pesticide accounted for at least 25 percent of the total imports into the country [40].

Another recent study shows glyphosate contamination in CKDu-affected areas [41]: the glyphosate levels of lakes were between 28 and 45 μg /L. Glyphosate was detected in all sediment samples (85–1000 μg/kg), and a strong linear relationship with the organic matter content was observed. Presence of high valence cations such as Fe3+ and Al3+ in topsoil resulted in the formation of glyphosate-metal complexes, resulting in the strong retention of glyphosate in soil. Rainfall is another potential source of exposure. A study based in Argentina found up to 67 μg/kg of glyphosate in 80% of collected rain samples [42].

Individuals in the CKDu-affected areas may also be ingesting more glyphosate through the consumption of locally grown food as well as imported foods such as red lentils. The 2016 Department of Census and Statistics Household Income and Expenditure Survey has estimated that the average consumption of red lentils was 583.40 g per person per month. Red lentils have become a staple part of the Sri Lankan diet almost similar to rice. Australia and Canada have become important suppliers of red lentils to Sri Lanka [43]. Tests conducted by the Canadian Food Inspection Agency (CFIA) on many samples of these lentils and moong dal grown by farmers in Canada and Australia found an average 282 parts per billion and 1000 parts per billion of glyphosate respectively, which is high by any standard [44,45].

Most of the research in Sri Lanka that has been conducted on CKDu during the past decade has resulted in multiple hypotheses. These hypotheses include arsenic, cadmium, nephrotoxic pesticides, fluoride, the use of cheap aluminum vessels, cyanobacteria, ayurvedic treatments, snake bites, and the use of nonsteroidal anti-inflammatory medications as possible causes of CKDu [36,46,47,48]. Unfortunately, these hypotheses were presented as possible alternatives to each other and not as complementary risk factors acting synergistically. This is a missed opportunity. 

Our work builds on the existing research on dehydration and pesticide exposure to explain the etiology of CKDu [49,50,51], while exploring the synergistic actions of two specific agricultural chemicals, glyphosate and paraquat, and their contribution to CKDu in Sri Lanka. 

Climate change has resulted in a significant rise in the global mean temperature and it is predicted to get worse [23]. This means more extreme heat events and heat exposure for people working in agriculture in the hot climates. A recent study of 192 agricultural workers in Florida showed that the odds of acute kidney injury increased 37% for each 5-degree (°F) increase in the heat index [52].

Sri Lankan agricultural workers are subjected to extreme heat and humid conditions with excessive perspiration. Though their dehydration status is unknown, numerous government and academic reports show that their well water is contaminated [24,37,49,53,54]. As a result, these farmers may ingest significant amounts of glyphosate, other pesticides, calcium, magnesium, fluoride, and other heavy metals with their drinking water during a normal workday [3,33]. As we show below, the ingestion of glyphosate along with other heavy metals and pesticides is likely an important factor in the causation of CKDu, due in part to glyphosate’s ability to inhibit the cytochrome P450 enzymes [55] and to chelate and transport metals to the kidneys [12,56].

## 3. The Web of Causation of CKDu 

**Figure 1:**
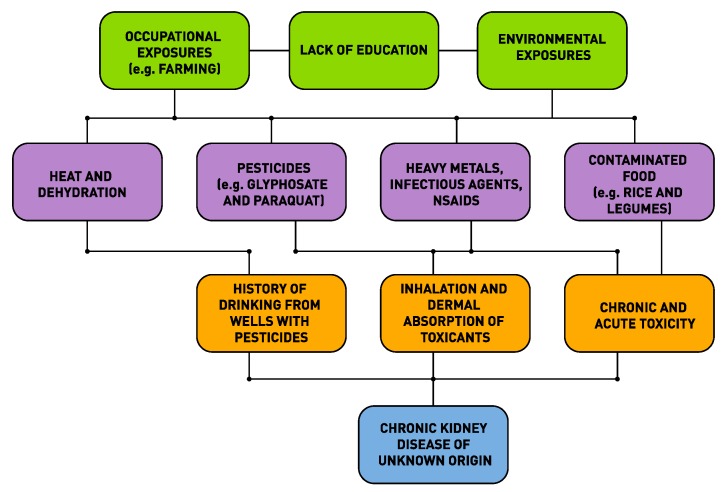
Web of causation of chronic kidney disease of unknown etiology (CKDu) in Sri Lanka.

As was first suggested by MacMahon and Pugh (1960) for non-communicable diseases (NCDs) including CKDu, there is no single factor responsible for their causation [57]. There are multiple factors that contribute to the etiology of an NCD and the role of each factor can be mapped out and their contribution to the disease burden could be measured. This was the origin of the concept of attributable risk in epidemiology and the use of this concept in program planning to design interventions to reduce the disease burden. The mapping out of these causal factors resulted in the concept of a “web” of causation. As an illustration, we have produced below a web of causation for CKDu based upon the current status of our knowledge (see Figure 1). 

In the remainder of this paper, we will first present the evidence from the research literature that glyphosate is capable of substituting for glycine erroneously during protein synthesis. We will then discuss several aspects of CKDu where one or more causative factors can be predicted to work synergistically with glyphosate to cause harm, due to glyphosate’s disruption of critical proteins involved in protection from these other toxic elements. We will conclude with a summary of our findings. 

## 4. Glyphosate as a Glycine Analogue 

Glyphosate can be characterized as an amino acid analogue of glycine, and much of its toxicity likely derives from this feature. Glyphosate has been demonstrated to interfere with enzymes that have glycine as a substrate, such as δ-aminolevulinic acid synthase, the catalyst for the first step in the synthesis of porphyrin rings, which are core components of both chlorophyll and heme [58]. Glycine is a ligand for N-methyl D-aspartate (NMDA) receptors in the brain, and glyphosate has been shown to activate NMDA receptors, causing neurotoxicity, likely also through its role as a glycine analogue [59]. Glyphosate is in fact a complete glycine molecule, except that the nitrogen atom is bound to an additional methylphosphonyl group. 

It has been proposed that glyphosate’s insidious cumulative mechanism of toxicity to humans may be realized through a unique ability to substitute for glycine, a coding amino acid, during protein synthesis [60]. It is possible to explain the strong correlations that are seen between the rise in a large number of debilitating chronic diseases and glyphosate usage on core crops through such a mechanism [61,62]. Detailed descriptions of strong glycine dependencies in proteins linked to various diseases have been presented in a sequence of recent papers, relating glyphosate substitution for glycine during protein synthesis as a plausible contributory cause of diabetes, obesity and Alzheimer’s disease [60], amyotrophic lateral sclerosis (ALS) [63], autism, multiple sclerosis and prion diseases [64], anencephaly [65], CKDu [66] and gout [67].

Monsanto researchers conducted a remarkable study in 1989 which provides strong evidence that glyphosate is getting incorporated into proteins by mistake in place of glycine [68]. In this study, bluegill sunfish were exposed to radiolabelled glyphosate and various tissue samples were examined for the presence of radiolabel, as an indicator of glyphosate accumulation in the tissues. They then used an assay to measure glyphosate levels in the same tissue samples and discovered that only up to 20% of the radiolabel could be accounted for as glyphosate. However, they found that, by subjecting the tissue samples to proteolysis by proteinase K, they could increase the yield of glyphosate up to 70%. They even used the words “incorporated into the protein” to explain the observed increase in yield, because proteolysis would be needed to break up the peptide sequence into individual amino acids, freeing up glyphosate so that it could be detected by standard assays. 

A strong case can be made for glyphosate substitution during protein synthesis through observations that have been noted concerning the unique aspects of glyphosate’s suppression of 5-enolpyruvylshikimate-3-phosphate (EPSP) synthase (EPSPS) in the shikimate pathway. This is believed to be the main mechanism of toxicity of glyphosate to weeds. Multiple species of plants and multiple species of microbes have independently acquired a mutated form of EPSPS that is completely insensitive to glyphosate, even at high concentrations [63,69], and this mutation forms the basis of the engineered gene in glyphosate-resistant core crops [70]. In all cases, the mutation involves disrupting a highly conserved glycine residue that forms part of the pocket where the substrate phosphoenol pyruvate (PEP) fits snugly.

A paper published in 1997 by Monsanto researchers pointed out several remarkable aspects of glyphosate’s interactions with EPSPS [71]. These authors proposed that glyphosate acts as an allosteric inhibitor of PEP binding. However, they observed several facts that make glyphosate’s behavior exceptional. Noting glyphosate’s uniqueness, they wrote: “To date no glyphosate analog or derivative has been identified that is more potent than glyphosate either as a herbicide or as an EPSPS inhibitor.” This statement is corroborated in another published paper, which stated that over a thousand different analogues of glyphosate with similar shape and biophysical properties were tested, yet none of them was nearly as effective as glyphosate in suppressing EPSPS activity [70,72]. 

The Monsanto researchers observed that various mutations in EPSPS have rather contradictory behavior in terms of their effects on PEP binding versus their effects on glyphosate inhibition [71]. Mutations that lose the conserved glycine residue at the PEP binding site have devastating and categorical effects on glyphosate but much weaker effects on PEP. They wrote: “For example, replacing the conserved glycine-101 with alanine (G101A) in petunia EPSPS weakens the Km(app) for PEP by 40-fold, reduces kcat by 2-fold, but perturbs glyphosate inhibition by nearly 5000-fold.” This would be expected if glyphosate works through substituting for glycine via the DNA code, which, with alanine substituted, no longer matches. 

On the other hand, other mutations that disrupt PEP binding had little effect on glyphosate inhibition. For example, in the case of a mutation, R104K, in Bacillus subtilis mutants, the enzyme became even more sensitive to glyphosate inhibition, yet its catalytic efficiency towards PEP dropped dramatically. Since lysine (K) is smaller than arginine (R), this stimulatory effect on glyphosate could make sense in terms of allowing additional room for the methylphosphonyl group on glyphosate’s nitrogen atom. A similar effect was observed for other mutations at the PEP binding site, as long as they did not involve substitutions for the glycine residue [71].

A seminal paper was published in 2018 by researchers from DowDuPont involving CRISPR technology to tweak the gene coding for EPSP synthase in maize [73]. It revealed important insights into the structure of the modified version that yielded the best result, in terms of high activity for PEP and high resistance to glyphosate. The optimized variant, called D2c-A5, had a G101A substitution as in the petunia plant above, which, as expected, made it completely insensitive to glyphosate. However, the extra methyl group created steric hindrance for PEP binding, but this could be corrected through additional mutations to nearby amino acids, substituting a smaller amino acid so as to expand the pocket to now fit PEP. As we would predict, as long as glycine is substituted, sensitivity to glyphosate is lost. 

It should be anticipated that mutations that impinge on the glycine residue might disturb glyphosate’s ability to fit into the peptide chain. As expected, by substituting leucine for proline at location 101, Staphylococcus aureus has discovered another clever way to protect EPSPS from glyphosate’s effects [74]. In this case, the bulkier leucine residue crowds the nearby glycine residue but does not perturb the binding site for PEP at all. Glyphosate is no longer able to substitute for glycine due to steric hindrance with leucine. Enzymatic activity towards PEP is maintained at a high level while glyphosate inhibition is disrupted. 

Perhaps the most remarkable study for supporting the idea that glyphosate inhibits EPSPS by substituting for its highly conserved glycine residue is one conducted on an *Escherichia coli* (*E. coli*) version of the enzyme where the critical glycine residue is located at position 96 [75]. In this study, G96 was mutated to serine, which resulted in a dramatic change to the protein function. Its normal reaction was completely disabled, but instead a reverse reaction became possible, where EPSP, the normal product, was converted back to shikimate-3-phosphate, releasing pyruvate. This G96S version of EPSPS was highly inhibited by shikimate-3-phosphate through product inhibition, but not sensitive at all to glyphosate inhibition, as expected because there is no glycine residue at the critical site in the enzyme for glyphosate to displace. 

One can now imagine the consequences of glyphosate substituting for G96 in E coli. It is likely that such a corrupted enzyme would behave similarly to the G96S mutation, driving the reaction in reverse. While only some percentage of the EPSPS molecules would be affected, they would work against production of EPSP by undoing the reaction product of the intact versions of the enzyme. The net result is a waste of the energy in the phosphate bond of PEP, used to produce the EPSP that is then reverted back to pyruvate and shikimate-3-phosphate by the G96-glyphosate substituted enzyme.

Glyphosate’s ability to substitute for a coding amino acid is not unique. There are several examples in the research literature of other toxic substances, many of which are naturally produced, which produce their toxic effects through amino acid substitution. One of these is glufosinate, a naturally produced amino acid analogue of glutamate that is currently gaining popularity as an alternative herbicide to handle glyphosate resistant weeds [76]. Several species of fine fescue release large amounts of 3-hydroxyphenylalanine, a non-coding amino acid analogue of phenylalanine (commonly known as meta-tyrosine) into the rhizosphere surrounding their root zone, which works as a cytotoxin (herbicide) to inhibit root growth of other species of plants. Researchers have suggested that m-tyrosine may be useful as a natural herbicide in crop management [77]. Other examples include Azetidine-2-carboxylic acid (Aze), an analogue of proline [78], β-Methylamino-l-alanine (BMAA), an analogue of serine [79,80], and L-canavanine, an amino acid analogue of arginine [81]. All of these amino acid analogues cause serious diseases, often with a long latency period, through the disruption of protein function of a diverse number of proteins. 

## 5. Glyphosate and Phosphate Binding 

We have formed the hypothesis that phosphate-binding sites of many proteins besides EPSP synthase may also be especially susceptible to glyphosate substitution for a highly conserved glycine residue at that site. There is a vast array of proteins that bind extremely important small phosphate-containing molecules, such as adenosine triphosphate (ATP), guanosine triphosphate (GTP), nicotinamide adenine dinucleotide phosphate (NADPH), pyridoxal-5′-phosphate (PLP), glucose-6-phosphate (G6P), flavin mononucleotide (FMN), flavin adenine dinucleotide (FAD), etc. It is insightful to study the features of phosphate binding sites of EPSP synthase and, more generally, of any protein that binds phosphate. 

Although most phosphate binding sites cannot be characterized by a specific motif, it is possible to use a statistical approach to capture features of phosphate binding sites in proteins. Such an approach was used successfully by a team of researchers who were specifically interested in ATP-binding sites [82]. They analyzed 168 nonredundant ATP protein binding chains, and, using a machine learning approach, produced compositional data characterizing which amino acids were preferentially associated with the phosphate binding site. They found that glycine, along with the three positively charged amino acids arginine, lysine and histidine, were statistically significantly more likely to be an ATP-interacting residue. They summarized: “It can be inferred that the Gly and positively charged amino acids are important for the interaction with ATP.” This is reasonable, because the positively charged amino acids can hydrogen bond to the negatively charged phosphate moiety to secure it in place, and glycine, being the smallest amino acid, provides flexibility as well as room for the phosphate anion. This model fits well for EPSP synthase. *E. coli*’s EPSP synthase has two lysine residues, as well as a histidine and an arginine residue, that are present at the PEP binding site [83]. It is likely that glyphosate’s methylphosphonyl group is well secured into the pocket intended for phosphate during protein synthesis, aided by electrostatic bonding to the neighboring positively charged amino acids. 

This principle likely carries over to phosphate binding associated with other phosphorylated ligands, such as pyridoxal 5′-phosphate (PLP, vitamin B6). For example, two glycine residues (Gly219 and Gly257) as well as an arginine (Arg258) and a histidine (His179) (both positively charged) are all involved in stabilizing binding to the phosphate moiety of PLP in the enzyme lysine decarboxylase [84]. Mitochondrial δ-aminolevulinic acid synthase (the rate limiting enzyme in pyrrole synthesis) requires pyridoxal phosphate as a cofactor [85], and it has a GxGxxG motif beginning at residue 274 (GAGAGG) and a glycine-rich sequence beginning at residue 417 (GLYGARGGG). It may be that glyphosate’s mechanism of inhibition of this enzyme [58] involves glycine substitution at the phosphate-binding site.

Kinases are an important class of enzymes that transfer a phosphate from ATP to the substrate. Their ATP-binding site has a characteristic glycine-rich motif, GxGxxG, where the middle glycine in particular is almost universally present and crucial for catalysis, because it coordinates the γ phosphate of the ATP molecule and facilitates phosphoryl transfer. An important study using oligonucleotide-directed mutagenesis showed that substitution of either of the first two glycines by serine or alanine had a profound effect on a kinase, increasing the rate of ATP hydrolysis but sharply reducing phosphoryl transfer to substrate. In other words, the energy in the ATP phosphate bond was wasted. This has parallels with the G96S substitution in EPSP synthase discussed above. 

Notably, a study on *E. coli* which looked at protein expression found that multiple ATP-binding site proteins were upregulated in the presence of glyphosate [86]. The data from the appendix of this paper on ATP-binding components are reproduced here as Table 1. It is likely that upregulation is a consequence of the fact that glyphosate is getting inserted into the protein at the ATP-binding site and disrupting its catalytic activity. Many of these ATP-binding sites involve transporters, and the ATP-binding site of bacterial transporters usually has the Walker A GxxGxGKS/T motif which would be highly susceptible to glyphosate disruption [87].

FAD has a pyrophosphate (PPi) moiety at its center, and studies on multiple enzymes that bind FAD have revealed a characteristic glycine-rich motif, GxGxxG, at the PPi-binding site. The second glycine, in particular, because of its missing side chain, permits close contact to oxygen atoms in the PPi of FAD [88]. Glyphosate substitution here may be responsible for the observed suppression of enzyme activity of succinate dehydrogenase in the mitochondrial Complex II, which binds FAD at the sequence GAGGAG [89]. 

Ribulose-1,5-bisphosphate carboxylase/oxygenase (RuBisCO) is arguably the most common protein on earth. It is involved in the first major step of carbon fixation in plants. Notably, it contains 22 completely conserved glycine residues [90], and it binds phosphate at three different sites [91]. Multiple studies have found that glyphosate suppresses this enzyme in plants. De Maria et al. found a 26% reduction in RuBisCO activity in the leaves of Lupinus albus one week after application of 10 mM glyphosate [92]. Picoli et al. found a reduction in the assimilation rate of CO2 in glyphosate-resistant ryegrass following exposure to glyphosate [93]. Zobiole et al. observed that glyphosate-treated glyphosate-resistant soybean plants become chlorotic (yellow) due to a decrease in the rate of photosynthesis [94].

## 6. Synergy between Glyphosate and Other Toxic Elements 

**Figure 2:**
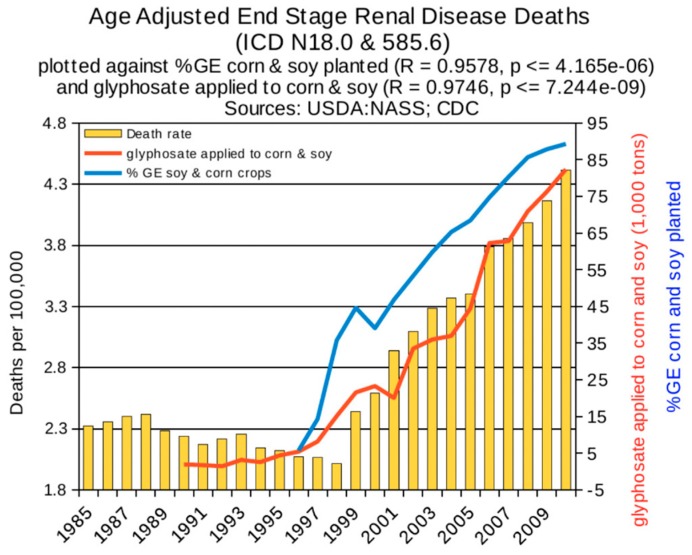
Correlation between age-adjusted End Stage Renal Disease deaths and glyphosate applications and percentage of US corn and soy crops that are GE. (From Swanson et al., 2014) [61].

**Figure 3:**
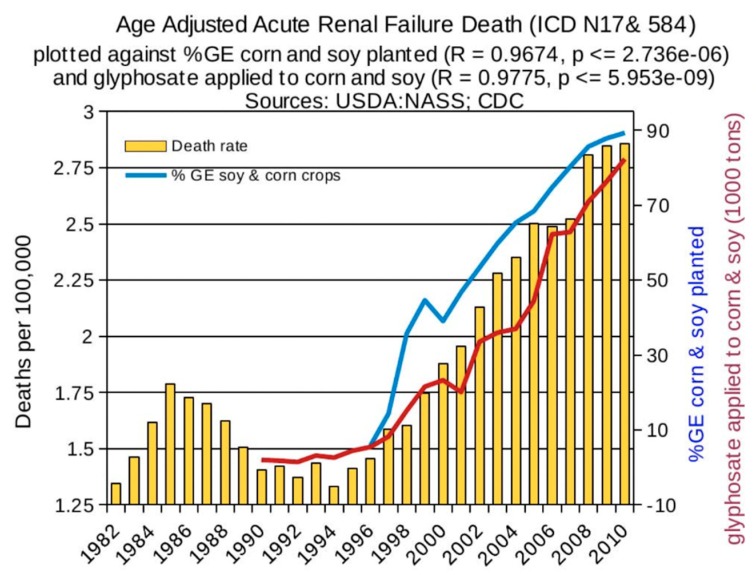
Correlation between age-adjusted renal failure deaths and glyphosate applications and percentage of US corn and soy crops that are GE. (From Swanson et al., 2014) [61].

**Figure 4:**
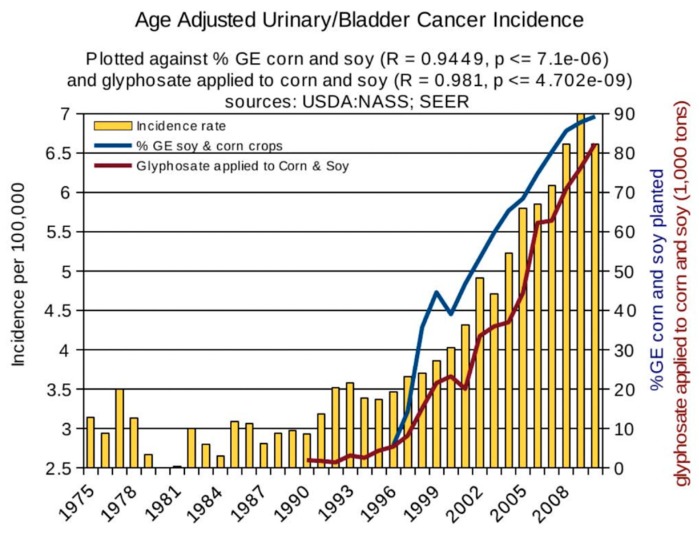
Correlation between age-adjusted bladder/urinary tract cancer and glyphosate applications and percentage of US corn and soy crops that are GE. (From Swanson et al., 2014) [61].

Statistical analyses of disease trends have shown that multiple chronic diseases are rising in incidence in the United States over the past two decades in step with the dramatic rise in the use of glyphosate on core crops. We reproduce here three figures from the seminal paper by Swanson et al. (2014) [61] showing correlations between the rise in glyphosate usage and end stage renal disease (Figure 2), deaths due to renal failure (Figure 3) and bladder cancer (Figure 4). In all cases, the p-value for the likelihood that the correlation could have occurred by chance is extremely small, with several zeros before the first significant digit.

In this section, we discuss how glyphosate could be expected to collaborate with other toxic exposures to increase susceptibility risk to CKDu. In some cases, we draw on the research literature on the specific roles of certain critical glycine residues in the proper function of proteins whose dysfunction is known to be linked to CKDu. First, it should be noted that glyphosate-based formulations have been shown to have powerful adverse effects on the kidneys in rat studies, even at ultra-low doses. A study published in 2015 involved a transcriptome analysis on liver and kidney gene expression following exposure to ultra-low doses of Roundup doses [95]. They found that Roundup induced alterations in gene expression of thousands of proteins, both upregulated and downregulated, in both liver and kidney. They noted that “Observed alterations in gene expression were consistent with fibrosis, necrosis, phospholipidosis, mitochondrial membrane dysfunction and ischemia, which correlate with and thus confirm observations of pathology made at an anatomical, histological and biochemical level.” They wrote in the conclusion: “The results of the study presented here indicate that consumption of far lower levels of a GBH formulation, at admissible glyphosate-equivalent concentrations, are associated with wide-scale alterations of the liver and kidney transcriptome that correlate with the observed signs of hepatic and kidney anatomorphological and biochemical pathological changes in these organs.” [95], p. 13.

In examining 46 biopsies of patients with CKDu, Laura Lopez-Marin et al. found interstitial fibrosis and tubular atrophy with or without inflammatory monocyte infiltration [8]. In addition, generalized sclerosis, increased glomerular size, collapse of some glomerular tufts, and lesions of extraglomerular blood vessels (such as intimal proliferation and thickening and vacuolization of the tunica media) were observed.

### 6.1. Glyphosate and Cytochrome P450 Impairment 

Cytochrome P450 (CYP) enzymes are responsible for metabolism of thousands of endogenous and exogenous chemicals in the liver. Defective CYP enzymes can lead to acute sensitivity to toxicity of various drugs and mycotoxins. CYP enzymes have been shown to play an important role in detoxification of Fusarium-produced T-2 toxin, an important mycotoxin [96]. 

In addition, P450 molecules are critical to the production of steroid hormones, Vitamin A and Vitamin D. Activated D is produced in the proximal renal tubule. It plays a fundamental role in several disease processes that are closely related to CKD, including inflammation [97]. Vitamin D may also play a role in racial differences in mortality seen in dialysis patients [98]. Several studies have reported that treatment with Vitamin D analogues, such as paricalcitol, results in an increase in the life expectancy of patients with renal failure [99] (and references therein). The three most important steps in vitamin D metabolism, 25-hydroxylation, 1α-hydroxylation, and 24-hydroxylation, are all performed by CYP enzymes [100].

The P450 family of CYP enzymes has a signature motif characterized as FGxGRHxCxG (also known as CXG), with two and often three highly conserved glycine residues [101]. This motif is located at the heme-binding iron center, and the synthesis of heme is also disrupted by glyphosate through its competitive inhibition of glycine as substrate to the reaction catalyzed by δ-aminolevulinic acid synthase, the rate-limiting step in synthesis of the component pyrrole ring [58]. Iron chelation by glyphosate may also impair iron bioavailability. CYP enzymes also depend on the cofactor, NADPH, which plays an essential role in splitting the oxygen dimer to add a single oxygen atom to the substrate. A highly conserved glycine residue forms a main-chain hydrogen bond with phosphate at the FMN-binding site of the accessory protein, cytochrome P450 reductase, which is necessary for catalytic activity of all CYP enzymes [102]. Both the CYP enzymes and CYP reductase have binding sites for NADP(H), a phosphorylated molecule. NAD(P)H is synthesized in the liver from tryptophan, a direct product of the shikimate pathway which glyphosate disrupts [103,104]. Hence, it comes as no surprise that glyphosate has been shown to highly suppress CYP enzyme activity in rat liver [105]. 

### 6.2. Glyphosate and Toxic Metals 

We have already discussed multiple papers mentioning toxic metals, particularly arsenic, as likely playing a role in CKDu. While phosphate fertilizers are a known source of toxic metals, glyphosate herbicide formulations are as well. A recent publication by Defarge et al. (2018) analyzed multiple formulants of glyphosate for levels of toxic metals [20]. Indeed, they found high levels of contamination of arsenic (As), chromium (Cr), nickel (Ni) and lead (Pb) in many of the formulations. Specifically, they wrote: “In total, all except 3 formulations had 5–53 times the permitted level of As in water in European Union or USA; all except 1 had Cr above (up to 40 times) the permitted level; all except 1 contained Ni, with 19 samples being above the permitted level (up to 62 times); 6 contained up to 11 times the permitted level of Pb.” [20] (p. 160). 

A paper by Jayasumana et al. published in 2015 provided evidence that glyphosate may be working synergistically with toxic metals to harm the kidneys in Sri Lankan agricultural workers [39]. Their study looked at urinary samples drawn from patients, controls living in the same region, and controls living elsewhere. While urinary levels of multiple toxic metals were generally higher in both patients and colocalized controls compared to controls outside of the endemic area, the most striking finding was that glyphosate excretion was very high in both endemic controls (39 times more) and patients (46 times more) compared to non-endemic controls [39].

Glyphosate, as a strong chelator, should be capable of transporting arsenic past the gut barrier and on to the kidneys, where it is being released in the acidic environment of the urine to cause stress to the renal tubules [11]. By analogy, it has also been proposed that glyphosate could be transporting aluminum to the brain, causing damage to the brainstem nuclei when aluminum is released in the acidic environment of the terminal watershed [106]. There is a precedent with citrate being known to carry aluminum across the gut barrier via a similar chelation mechanism [107]. Aluminum from sub-standard utensils used in cooking could be contributing to CKDu in Sri Lanka, especially when combined with acidic cooking conditions and fluoride stress [108].

### 6.3. Hyperphosphorylation and Tubular Fibrosis Following Injury 

Tubulointerstitial fibrosis is a core feature of CKDu in Sri Lanka. Interstitial fibrosis and tubular atrophy were the dominant histopathological observations in a study of 64 CKDu patients from North Central Sri Lanka [109,110]. This condition is characterized by the overproduction of extracellular matrix by infiltrating fibroblasts, invoked as a response to injury. Renal cells damaged by injury or oxidative stress release cytokines and growth factors such as transforming growth factor-β (TGF-β), endothelial growth factor (EGF) and platelet derived growth factor (PDGF) [111,112]. The receptors for these signaling molecules are typically protein kinases, belonging to one of two broad classes: serine/threonine kinases and tyrosine kinases. These receptors are activated through phosphorylation of multiple serine, threonine or tyrosine residues, and the cascade that is launched can include autophosphorylation in a positive feedback loop. The resulting signal transduction pathways lead eventually to nuclear activation of expression of proteins involved in matrix production, inducing fibrosis [113]. 

TGF-β is well established as a cytokine linked to renal tubular fibrosis [113,114]. The TGF-β receptor is a serine/threonine kinase that is activated through phosphorylation of its own serine and threonine residues. In particular, there is a motif referred to as a “GS domain” that contains the sequence 185Thr–Thr–Ser–Gly–Ser–Gly–Ser–Gly192, where all of the serine and threonine residues are potential phosphorylation sites [115]. The four glycine residues within this sequence are obviously potential sites for glyphosate substitution. Biophysical arguments support the idea that the methyl-phosphonyl group of glyphosate would be a very strong mimetic of serine phosphorylation. It has been demonstrated experimentally that aspartate and glutamate can both act as phosphoserine mimetics, and this property has been exploited in experiments that induce a constitutive psuedophosphorylation of serine through substitution by glutamate or aspartate [116]. Glyphosate, similarly negatively charged and even more closely matched (methylphosphonate vs phosphate), should act in a similar way. 

Experiments have shown that substitution of a negatively charged amino acid, such as aspartate or glutamate, in place of a serine residue, can yield a behavior that is very similar to that of a phosphorylated serine residue, causing the protein to become constitutionally activated. A zebrafish version of an enzyme that is normally activated through serine phosphorylation is missing the serine residue, yet it behaves as if it is permanently serine phosphorylated. An analysis of the enzyme led to the conclusion that its novel aspartate residue two codons away from the missing serine was providing the negative charge that maintained it in an activated state [117]. 

Serine/threonine phosphatases terminate TGF-β signaling [118]. Dephosphorylation of phosphorylated serines and threonines is likely to be problematic with glyphosate substituting for glycine. Serine/threonine phosphatases have multiple glycine-containing signature motifs that are involved in metal coordination and phosphate binding, including GDxHG, GDxVDRG, GNHE, HGG, and RG [119]. Thus, both pseudophosphorylation through glyphosate substitution for glycines adjacent to serines as well as impaired phosphatase activity due to glyphosate substitution for critical glycines in phosphatases can be expected to drive the cellular signaling towards excessive phosphorylation, systemically, leading to the nuclear activation of synthesis of proteins involved in fibrosis. 

Multiple receptor tyrosine kinases (RTKs) play a crucial role in the development of renal fibrosis as well [120]. Receptors for epidermal growth factor, fibroblast growth factor, and vascular endothelial growth factor are all examples of receptor tyrosine kinases involved in the disease process [120]. A number of drugs used to treat cancer, degenerative diseases, and cardiovascular diseases work as inhibitors of tyrosine kinases. At least two of these inhibitors, Nintedanib [121] and Suramin [122], have been shown to attenuate renal fibrosis in CKD. 

Phosphotyrosines are normally aggressively dephosphorylated by phosphatase enzymes, but these are likely to be severely disabled by glyphosate substitution. The central glycine residue in the tyrosine phosphatase signature motif, (H/V)CxxGxxR(S/T), is highly conserved. Substitution of proline or alanine for the conserved Gly-127 residue within this motif in T-cell protein tyrosine phosphatase resulted in a 400-fold decrease in catalytic activity [123]. Thus, the processes that remove phosphates from tyrosine residues are likely to be impeded by glyphosate as well, resulting in systemic hyperphosphorylation of tyrosines.

### 6.4. Paraquat and MATE1

According to the U.S. Centers for Disease Control and Prevention (CDC), paraquat is one of the most commonly used herbicides in the world. In Sri Lanka, paraquat was used extensively in the rice paddies in the 1980s to control weeds. By the mid-1990s, glyphosate became a popular alternative, mostly because paraquat’s acute toxicity was becoming much more appreciated and glyphosate was considered a safer alternative. CKDu was first recognized in Sri Lanka in the mid-1990s, when both paraquat and glyphosate were in active use. 

Although paraquat has been banned by the European Union since 2007, hundreds of countries continue to use it, including El Salvador, where it has the highest sales among pesticides [124]. Paraquat and glyphosate are both popular as post-emergence herbicides for sugarcane [125], and glyphosate is the herbicide of choice for ripening the cane just before harvest [126]. El Salvador is experiencing an alarming rate of kidney failure among the sugarcane workers [53]. Paraquat was phased out in Sri Lanka beginning in 2008 due to concerns over its use for self-harm, and glyphosate was banned beginning in 2016, although the ban was lifted in 2018. However, both formulations continue to be available on the black market.

Paraquat (1,1′-dimethyl-4,4′-bipyridinium dichloride) is actively secreted in the kidney via the proximal tubules, which are the target site of CKDu. Paraquat induces reactive oxygen species (ROS), including superoxide, hydrogen peroxide, and the hydroxyl radical, ultimately damaging the proximal tubules [127]. These ROS cause DNA damage and genotoxicity, as well as destroying membrane lipids and inducing cell death [128]. An in vitro study on rabbit renal tubules demonstrated that paraquat appears to disrupt the mitochondrial electron transport chain, inducing oxidative stress and depleting glutathione and energy supplies [127]. Like the mushroom-derived nephrotoxin orellanine, also a bipyridinium, paraquat selectively targets renal tubular epithelial cells [129,130,131,132]. Damage to proximal tubules could mean reduced elimination and more toxicity for other organs [133].

In addition to specifically targeting the renal proximal tubules, there is good evidence to support the idea that paraquat is synergistically toxic with glyphosate, based on the argument that glyphosate can substitute for glycine during protein synthesis. The main protein responsible for the export of paraquat from the apical membrane of tubular cells into the tubule lumina for excretion into the urine is called Multidrug and Toxin Extrusion 1 (MATE1). Designer mice with a deficiency in MATE1 are especially susceptible to renal paraquat toxicity, due to a much higher accumulation within the tubular cells [128]. These authors wrote: “Our data indicated that MATE1 played a critical role in the renal elimination of PQ [paraquat] and disruption of MATE1 function remarkably potentiated PQ nephrotoxicity in mice.” [128] (p. 2477). 

Gene sequence alignments of MATE1 from four species reveals 32 glycine residues that are conserved among all four species, as well as another 6 that are conserved among 3 out of 4 [134]. In a study on observed human single-nucleotide polymorphisms (SNPs), a glycine mutation (G64D) in MATE1 and another glycine mutation (G211V) in a related protein, MATE2-K, both caused transport activity to be completely abolished [135]. This would result in the accumulation of toxins such as paraquat in the tubules. Glyphosate substitution for these critical glycines, as well as any of the other highly conserved glycines, would likely also severely disrupt protein function. 

Multidrug resistance protein 1 (MDR1), also known as p-glycoprotein, is another efflux protein expressed in the kidneys that has also been shown to be protective against paraquat toxicity [136,137]. This protein binds to ATP and has two nucleotide binding sequences matching the GxxGxG motif (GNSGCG beginning at residue 427 and GSSGCG beginning at residue 1070). ATP hydrolysis is coupled to binding and translocation of substrates across the membrane [138]. Thus, it is likely susceptible to disruption by glyphosate at the phosphate binding sites.

### 6.5. Impaired Antioxidant Defenses 

A study on gene expression in association with CKDu determined that proteins related to protection from oxidation damage are upregulated in association with CKDu, including glucose-6-phosphate dehydrogenase (G6PD), the rate-limiting enzyme in the pathway that regenerates the antioxidant NADPH, as well as enzymes involved in the synthesis of glutathione and the restoration of the reduced form of glutathione [139]. NADPH is needed to restore glutathione to its reduced (protective) state. The overexpression of these enzymes is an indicator that patients with CKDu are chronically exposed to oxidizing agents from the environment. 

Glutathione (GSH) is an essential antioxidant tripeptide, as it can protect from oxidative damage through reversible oxidation to the dimer form GSSG. Glutathione deficiency increases susceptibility to mycotoxins [140]. Glutathione is composed of glycine, glutamate and cysteine. It is possible that glyphosate disrupts glutathione by directly substituting for its glycine residue, as proposed in Seneff et al., (2017) [67]. Gamma glutamyl transpeptidase (GGT) is an enzyme that breaks glutathione down into its individual amino acids, allegedly so that the components can be distributed in the blood for reuptake and reassembly back into glutathione by another cell. However, another motivation for disassembly/reassembly might be that it is a defective version of the tripeptide due to glyphosate substitution for the glycine residue. Experiments involving exposing rat testes to Roundup demonstrated the upregulation of multiple enzymes involved in glutathione metabolism, including GGT, as well as the depletion of glutathione levels in the testes [141]. Notably, GGT is upregulated in association with end-stage chronic renal failure [142], and elevated GGT is an independent predictor of mortality in patients with stage 4–5 kidney failure [143]. 

Glutathione S-transferase (GST) plays an important role in liver detoxification of a large number of hydrophobic compounds by catalyzing their conjugation to glutathione, thus increasing their water solubility. Biliary excretion of glutathione conjugates of aflatoxin are a key mechanism for liver clearance of this mycotoxin [144]. Gly-146 is one of few residues that are strictly conserved in the GST superfamily. This residue lies within a conserved folding module called Motif II which maintains an internal hydrogen bond network in the face of heat stress, supporting protein stability under elevated temperature conditions. Substitution of either alanine or valine for this glycine destabilizes a conserved loop-helix substructure that is essential for proper folding [145].

Glucose-6-phosphate dehydrogenase (G6PD) is an important antioxidant enzyme, particularly in red blood cells, which regenerates NADPH, needed for glutathione reduction. Thus, it plays an essential role in maintaining antioxidant defenses. A study examining a possible association between G6PD deficiency and CKDu found that 20% of CKDu patients in Sri Lanka were deficient compared to only 2% of controls [1].

G6PD has three regions where it binds to molecules containing phosphate. One involves its substrate, glucose-6-phosphate. Another binds to the NADP+ molecule that will ultimately be reduced to NADPH. The third site is a region where it binds tightly to another NADP+ molecule, a site that has been dubbed the “structural NADP+ binding site” as opposed to the “catalytic NADP+ binding site” [146]. G6PD has multiple critical dependencies on glycines, mostly linked to these phosphate-binding sites. Highly conserved sequences within G6PD include a 9-residue sequence containing a glycine residue at the glucose-6-phosphate binding site, a GxxGDLA “nucleotide-binding” fingerprint at the catalytic NADPH binding site, and another EKPxG motif near the substrate binding site [147]. A glycine residue at location 488 is within the structural NADP+ binding site.

Glyphosate present in the serum could be readily taken up by RBCs and incorporated into G6PD in place of these critical glycine residues, disrupting enzyme activity. G6PD is one of the most highly mutated enzymes in humans. Mutations to a bulkier amino acid have been shown to decrease the binding affinity to NADP+ [146]. A glycine-to-arginine mutation in G6PD (G447R) leads to very low residual activity, resulting in a severe chronic hemolytic anemia [148]. A G274K mutation also leads to a defective version of the enzyme [149]. 

Gao et al. (2019) [150] use a cell culture model to show that the kidney is damaged by glyphosate-based herbicides and that the renal proximal tubule may be a main target. Though the toxicity of commercial formulations is well established, the authors demonstrate that glyphosate, the active ingredient of glyphosate-based herbicides, injures renal tubule epithelial cells via the NMDAR1/calcium influx/oxidative stress pathway, both in vitro and in vivo. They state that their findings “provide a theoretical basis and reference data to assess the risk of glyphosate and to explore the etiology of CKDu.” 

### 6.6. Aquaporins and Dehydration 

Aquaporins are a set of integral membrane proteins that form pores in the membrane to facilitate the transport of water through the membrane [151]. Aquaporins are especially important in the renal tubules, where they mediate osmotic water transport across the renal medullary epithelium, in order to concentrate the urine and protect from water loss. Aquaporin expression is upregulated in response to dehydration, mediated by vasopressin [152]. NSAIDS, such as Ibuprofen, have been shown to decrease aquaporin 2 expression in water-restricted rats [153]. It has been suggested that NSAID use may be a causal factor in CKDu, due to excessive loss of water through urine. Nocturia is a common early symptom of CKDu. A study on women with CKDu in an agricultural community in El Salvador showed that 40% of them took NSAIDS, and half of them suffered from nocturia [154]. 

Glyphosate may also cause excessive loss of water through urine, through substitution for certain critical glycine residues in aquaporin, as argued in Seneff and Orlando (2018) [66]. Most aquaporins contain two highly conserved glycine residues: Gly-57 in transmembrane helix (TM) 2 and Gly-173 in TM5 at the contact point where the two helices cross in human Aquaporin 1 [155]. Aquaporin 6 has an asparagine residue in place of the usual Gly-57, and this aquaporin functions not as a water channel but rather as an ion channel. This implies that substituting glyphosate for Gly-57 in other aquaporins would greatly disrupt their function in retaining water. Replacing this asparagine residue in Aquaporin 6 with glycine converts it back into a water channel, and replacement of the glycine at this site in Aquaporins 0, 1 and 2 completely blocked their expression as a water transporter [155]. 

Glyphosate-resistant soybean plants treated with glyphosate are more sensitive to drought and less efficient in converting water into biomass compared to those not exposed to glyphosate. Zobiole et al. (2010) [94] found a highly significant nearly perfectly linear relationship between the amount of glyphosate applied in a single treatment and the amount of water absorbed by 58 days post emergence. They hypothesized that disruption of aquaporin function could be the cause, although they admitted that the exact mechanism was unknown.

### 6.7. Cyanobacteria and Glyphosate 

Toxigenic cyanobacteria present a growing problem in Sri Lanka’s waterways, particularly in the North Central province where CKDu is endemic [156]. It is clear that their overgrowth is related to human activities, particularly in agricultural areas, where agricultural runoff of phosphate and nitrate fertilizers are supporting their growth. It is likely that glyphosate is also a major contributing factor. Glyphosate does not kill cyanobacteria, though it can kill beneficial species in waterways. The cyanobacteria are actually able to break the difficult C-P bond in glyphosate and fully metabolize it, utilizing the phosphorus atom in glyphosate as a source of phosphorus [157]. Drzyzga and Lipok [158] demonstrate that glyphosate is a significant factor for the phosphorus utilization strategy of cyanobacteria. 

Cyanobacteria produce multiple toxins that are known to be nephrotoxic, causing proximal tubule epithelial cell necrosis, which leads to the accumulation of cellular debris in the distal tubules [159]. 

### 6.8. Mycotoxins and Sulfotransferases 

It is possible that toxic metabolites produced by various species of fungi play a contributory role in CKDu. Fusarium mycotoxins, such as fumonisin B1 (FB1), deoxynivalenol (DON) and zearalenone (ZEA), are the most frequently occurring mycotoxins worldwide, and are common contaminants in food sources such as wheat. Studies on rats have shown that these metabolites are toxic to both the liver and the kidney, mainly through the induction of oxidation damage to membrane lipids [160]. In a study of 31 patients in the North Central province of Sri Lanka suffering from CKDu, aflatoxins, ochratoxins, and fumonisins were detected in 61.29, 93.5, and 19.4% of the patients respectively [161]. Aflatoxin (AF), zearalenone (ZEA) and deoxynivalenol (DON) are three of the most common toxic metabolites of fungi. 

Both DON and ZEA are capable of being sulfonated by sulfotransferase enzymes, which produce a metabolite that is more soluble and significantly less toxic than the original toxin. For example, it has been demonstrated that Aspergillus species are able to detox ZEA through conjugation with sulfate [162], and that wheat plants infected with Fusarium can produce multiple sulfated conjugates of DON. Don-15-sulfate was about 44-fold less inhibitory than native toxin, and no toxicity was observed for DON-3-sulfate [163]. It is possible that glyphosate is either killing the Aspergillus species or disrupting the synthesis of sulfated metabolites, both of which would accelerate the production of mycotoxins in soil. For example, the application of glyphosate-based herbicides at a dose 100 times lower than that used in agriculture is lethal to the fungus Aspergillus nidulans [164]. Glyphosate may disrupt the synthesis of sulfated metabolites because sulfotransferase is dependent on two highly conserved glycine residues in a GXXGXXK motif at the active site where PAPS, the activated sulfonate donor, binds to the enzyme [165]. The liver in humans also detoxifies multiple xenobiotics through sulfonation, producing a more soluble and less toxic product that can then be more readily cleared. The liver is very vulnerable to glyphosate toxicity [95]. One reason may be the disruption of sulfation pathways. 

## 7. Conclusions

**Figure 5:**
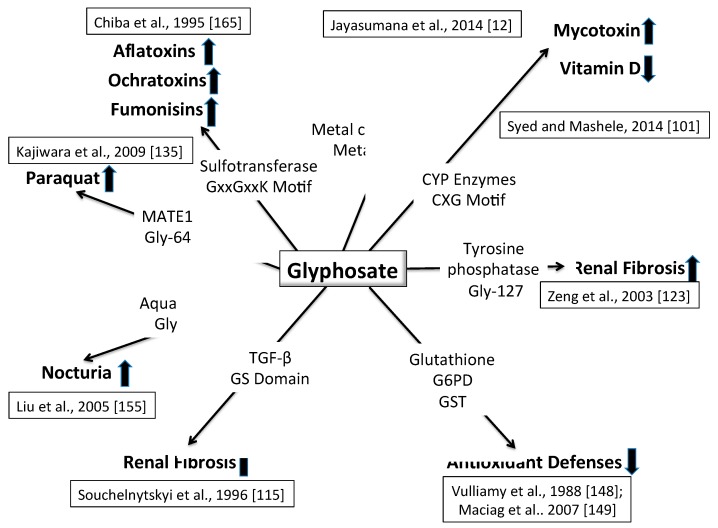
Schematic of multiple ways that glyphosate could cause kidney disease, working in concert with multiple other toxic exposures. See text for details.

As it becomes increasingly clear that CKDu is a multifactorial disease, the complexity of possibilities can be narrowed by an understanding of local environmental health conditions and resulting disease processes in the people that live and work there. A web of causality can begin to show related factors and research paths. We have proposed a plausible mechanism of toxicity for glyphosate that involves substitution for the coding amino acid glycine during protein synthesis, and we have shown theoretically how this could lead to increased toxicity of multiple other factors associated with CKDu. Figure 5 shows a schematic summarizing various synergistic effects of glyphosate with other chemical exposures, especially if it is indeed substituting for glycine. 

The causality outlined in this paper shows that one of the most important steps to take to reduce the burden of CKDu would be to stop the commercial sale of paraquat and glyphosate and end the black-market availability of these agricultural chemicals in Sri Lanka. This may be difficult to do in practice, but research is sorely needed to come up with viable alternatives that provide effective low-cost weed control while minimizing non-target toxic chemical exposures. 

Agricultural methods and practices that enhance crop productivity without subsequent damage to agricultural ecosystems and human health are well documented. These methods and practices have variations based on crops, culture, climate, and a number of other factors, but they share several core principles: the enhancement of soil health, recycling of biomass, utilization of cover crops, biodiversity above and below-ground, and an ecological focus on interactions and synergisms that benefit both plants and people. At least 31 million hectares (77 million acres) of farmland worldwide that utilize some of these principles is managed without agricultural chemicals [166].

One ecosystem approach is to replace chemical-based nitrogen and phosphate fertilizers with organic fertilizers in crop production, while improving soil health. This would, for example, help reduce water pollution and prevent the overgrowth of toxin-producing cyanobacteria [156]. Switching from chemical-based phosphate fertilizers to organic fertilizers would also help reduce arsenic exposure [167]. 

There are other measures that can be taken to reduce the likelihood of CKDu. Workers can be supported, through education and improved access, in wearing protective gear when using agricultural chemicals to minimize exposure risk. Government agencies and other institutions can conduct environmental and bio-monitoring in CKDu-affected areas to better understand local factors in the web of causation. Efforts to treat contaminated water with reverse osmosis filters could be expanded [167]. With regard to the treatment of CKDu, it is beyond the scope of this paper to discuss specific drugs and protocols. Still, it is clear that emphasis should be placed on preventive measures, especially since sophisticated treatment options such as dialysis and kidney replacement are often not realistic options for the agricultural workers due to the lack of access and prohibitive cost.

Our work shows the possible synergistic effects, specifically of glyphosate, on several metabolic processes in the presence of other agricultural toxicants, such as paraquat, along with adverse environmental and occupational conditions, such as hot climates, rising temperatures, and dehydration among agricultural workers, that can result in renal disease described by CKDu. Further exploration into these factors working together to cause CKDu is warranted.

## Figures and Tables

**Table 1:** ATP-binding proteins upregulated by E coli in response to glyphosate exposure. Reproduced from Lu et al., 2013 [86].

Protein	Fold Increased
D,D-dipeptide permease system, ATP-binding component	2.83
ATP-binding protein of nickel transport system	2.24
ATP-binding component of transport system for glycine, betaine and proline	12.96
Fused D-allose transporter subunits of ABC superfamily: ATP-binding components	2.03
ATP-binding component of transport system for maltose	2.38
Putative ATP-binding sugar transporter	2.10
Flagellum-specific ATP synthase	2.07
Putative ATP-binding component of a transport system	3.04
Putative part of putative ATP-binding component of a transport system	2.31
Putative ATP-binding component of a transport system	2.30
